# Association of plasma folic acid, vitamin-B12 and homocysteine with recurrent pregnancy loss. “A case control study”

**DOI:** 10.12669/pjms.39.5.7432

**Published:** 2023

**Authors:** Erum Afaq, Anwar Ali, Rabia Jamil, Hira Fatima Waseem

**Affiliations:** 1 Erum Afaq, M.Phil. Department of Physiology, Dow University of Health Sciences, Karachi, Pakistan, University of Karachi, Pakistan; 2 Anwar Ali, Ph.D. Department of Physiology, University of Karachi, Pakistan. University of Karachi, Pakistan; 3 Rabia Jamil, FCPS. Department of Obstetrics and Gynaecology, Dow University of Health Sciences, Karachi, Pakistan; 4 Hira Fatima Waseem, Ph.D. School of Public Health, Dow University of Health Sciences, Karachi, Pakistan

**Keywords:** Recurrent pregnancy loss, Homocysteine, Vitamin-B12, Folic acid

## Abstract

**Objective:**

To investigate the relationship of serum homocysteine, folic acid, and vitamin-B12 levels with recurrent pregnancy loss (RPL).

**Methods:**

A case-control study was conducted in the Department of Gynecology and Obstetrics, Dr. Ruth Pfau Civil Hospital, Karachi from July 2021 to June 2022. Total 124 participants were recruited from gynecology outpatient department after taking informed consent. The participants included 62 non-pregnant females with two or more consecutive unexplained RPLs and 62 healthy women having at least two successful deliveries without any pregnancy loss. Serum folic acid and vitamin-B12 levels were measured by chemiluminescent method and serum homocysteine was measured by enzyme-linked immunosorbent assay (ELISA). Comparison of quantitative variables with RPL cases and control was done by Mann-Whitney U-test (for non-normally distributed data) and independent sample t-test (for normally distributed data), while Pearson’s chi-square test was used for the association of qualitative variables with RPL cases and control. Correlation of homocysteine with vitamin-B12 and folic acid was assessed in RPL cases.

**Results:**

The median age of the study population was 27 (IQR 25-32) years. The median body mass index (BMI) was 26.25 (IQR 22-29) kg/m^2^. Cases had significantly lower serum folic acid (p-value=0.022), while vitamin-B12 was decreased in cases as compared to controls (p-value=0.295). Mean concentration of serum homocysteine was higher in RPL cases as compared to controls (p-value=0.094). There was significant association of serum vitamin B12 (p-value=0.001) and folic acid levels (p-value=0.004) with RPL. The homocysteine was not significantly correlated with vitamin-B12 (r=0.124, p=0.338) and folic acid (r=0.067, p=0.606) in the RPL group.

**Conclusion:**

Reduced level of serum folic acid and vitamin B12 was significantly associated with RPL cases, while homocysteine was marginally raised however the difference was not significant. Folic acid and vitamin B12 supplementation before next pregnancy in RPL patients is likely to be beneficial in improving pregnancy outcomes.

## INTRODUCTION

Recurrent pregnancy loss (RPL) is defined as two or more successive miscarriages before 20 weeks of gestation. It is a common obstetric complication. Estimated prevalence of RPL worldwide is one to three percent in women of reproductive age group.^1^ Many social, environmental and clinical factors contribute to RPL. A few causes of RPL that are of significant concern are genetic disorders, uterine pathologies, autoimmune diseases, endocrine dysfunctions and inherited and acquired thrombophilia. Maternal obesity and cigarette smoking are also important risk factors that increase the chances of miscarriage and stillbirth in pregnancy.^2^ Interestingly, a known etiology is found only in fifty percent of patients presenting with RPL, while the remaining cases remain unexplained.^3^

Hyperhomocysteinemia has been identified as one of the major contributory factors in the pathogenesis of RPL and its role has garnered a great deal of attention.^4^ Homocysteine, a sulfur-containing amino acid, is not present in human diet; rather, it is endogenously synthesized as an intermediate in methionine metabolism.^4^ It is also formed intracellularly through the demethylation of nutritional methionine during deoxyribonucleic acid or/and ribonucleic acid methylation. The two metabolic pathways that control plasma homocysteine levels are remethylation to methionine and transsulfuration to cysteine. There are two alternative pathways for the remethylation of homocysteine in humans, with the predominant one being vitamin-B12 dependent and connected to the folate cycle.^5^

Hyperhomocysteinemia can be caused by various acquired and genetic factors. The regulation of homocysteine requires several co-factors (including folic acid, vitamin-B6 and B12) and enzymes, such as methionine synthase (MS), methylenetetrahydrofolate reductase (MTHFR), and cystathionine beta-synthase (CBS).^6^ Vitamin-B12 and folic acid are essential for cell metabolism and any deficiency of these vitamins may cause interference in DNA synthesis and homocysteine metabolism.^6^,^7^ Disturbances in any step of the homocysteine metabolic pathway, caused by either vitamin deficiencies or enzymatic errors, can result in the accumulation of plasma homocysteine. Abnormal increases in plasma homocysteine levels can also be caused by defects in genes involved in the metabolism of folate, such as MTHFR gene.^6^

Increased plasma levels of homocysteine act as a vascular toxin that may lead to endothelial injury. It is an independent cause for the atherogenic and thrombotic constituents of arterial systems and an emerging risk factor for adverse pregnancy outcomes. Hyperhomocysteinemia causes damage to endovascular trophoblasts at the tips of spiral arteries, syncytiotrophoblasts, superficial/glandular epithelial cells of decidua, decidual/sertoli cells and endothelial lining of spiral veins, ultimately resulting in inflammation of the placenta. All these events lead to impaired perfusion and death of the fetus.^7^

During normal pregnancy, homocysteine levels tend to decline. Multiple factors contribute in reducing homocysteine levels during pregnancy such as increased glomerular filtration rate, hemodilution and diversion of homocysteine towards the fetus. Moreover, a healthy lifestyle and adequate dietary intake of vitamins, especially vitamin-B12 and folic acid, also helps in lowering homocysteine levels in the blood.^8^

Previous studies have shown that deficiency of vitamin-B12 and folate, along with hyperhomocysteinemia may lead to threatened pregnancy. Several studies have been undertaken in this regard in different geographical populations, but have reported significant variations in results. Since no study has yet been conducted involving Pakistani population, therefore, we designed our study to investigate the relationship of serum homocysteine, folic acid and vitamin B12 levels with RPL. Our study focuses on identifying factors that may contribute to RPL but have not been previously reported in Pakistan.

## METHODS

This case-control study was carried out in the Physiology department, University of Karachi and the Department of Gynecology and Obstetrics, Dr. Ruth Pfau Civil Hospital Karachi. Study population was recruited using nonprobability purposive sampling technique, from July 2021 to June 2022 after approval from Institutional Review Board (IRB-2175/DUHS/Approval/2021/652), Dow University of Health Sciences (DUHS) and Board of Advanced Scientific Research (ASRB/No./05796./Sc.), University of Karachi. Sample size was determined using Open-Epi online software. A sample size of 62 was calculated using mean homocysteine levels, 6.58±1.84 for cases and 5.5±0.8 for controls by keeping 95% power and 1% level of significance.^9^

All cases included in this study were between 18 - 50 years of age and had experienced at least two consecutive unexplained recurrent pregnancy losses before 20 weeks of gestation. They had presented to the Gynecology OPD for the treatment of RPL and expressed their willingness to participate in the study. Age-matched controls who had two or more normal term deliveries, no history of abortion or any other comorbidities were also included in the research, they had presented to the gynecology OPD and gave their consent to participate in the study. We excluded all cases of miscarriage due to anatomic causes, any autoimmune causes (systemic lupus erythematosus and antiphospholipid antibody syndrome), endocrine abnormalities (hypothyroidism, diabetes mellitus or hyperprolactinemia), smokers (present smoking or smoking within the last 10 years) and patients on vitamin-B12 and folic acid supplementation from our study.

A detailed medical history was obtained on pre-designed proforma. A thorough physical and medical examination was performed including the demographic profile and anthropometric measurements (height and weight for BMI). JNC Report 2003 was used for confirmation of hypertension. Written informed consent was taken from all participants before data collection.

Total 10ml of blood was sampled after overnight fasting (only water was allowed). 6ml was used for biochemical estimation of homocysteine, vitamin-B12 and folic acid. Blood serum was separated immediately and stored for further analysis. Remaining 4ml of blood was used to measure anti-cardiolipin antibody and lupus anticoagulant antibody to exclude acquired thrombophilia. Folic acid and vitamin-B12 levels were measured by chemiluminescent method using Roche Cobas e411 analyzer. Homocysteine, lupus antibody, cardiolipin IgG and IgM were measured by an enzyme-linked immunosorbent assay (ELISA) using Rayto RT-6000 Microplate reader. Reference ranges in our laboratory were as follows: 3.7-13.9 μ mol/L for homocysteine, 174-878 pg/ml for vitamin-B12 and 2.6-12.7 ng/ml for serum folate. Samples having lupus antibody >20IU/L and cardiolipin IgG and IgM >15 mol/L were excluded.

### Statistical Analysis

The statistical analysis was carried out using SPSS statistical software, version 22.0. The normality of continuous variables was checked using Shapiro-wilk’s test. Continuous variables were calculated as the mean± standard deviation (SD) for normally distributed data and median [Interquartile range (IQR): Q1-Q3] for non-normally distributed data. Frequency and percentages were calculated for categorical variables. Independent sample t-test was used to assess the mean difference of homocysteine in RPL patient’s and control. Mann-Whitney U-test was used to assess the mean difference in age, BMI, Vitamin-B12, and folic acid in RPL patient’s verses control.

Pearson’s chi-square and Fisher’s exact tests were performed to assess the association of age, BMI, homocysteine, vitamin-B12 and folic acid with RPL patients. Correlation between homocysteine with folic acid and vitamin-B12 in RPL group was assessed using the Spearman’s correlation test (non-parametric). p-value ≤ 0.05 was calculated as statistically significant.

## RESULTS

A total of 62 cases, along with equal number of healthy controls, fulfilling the inclusion criteria were selected for the study. Median age of the study population was 27 (IQR 25-32) years. Median body mass index (BMI) was 26.25 (IQR 22-29) kg/m_2_.

We found no significant differences in median age, BMI, vitamin-B12 and homocysteine levels between cases and controls (p-value>0.05). However, the median serum folate levels were significantly lower in cases 6.5 (IQR 3.5-9.4) ng/ml as compared to controls 8.1 (IQR 6.3-9.3) ng/ml (p-value<0.05) (Table-I).

**Table-I:** Comparison of baseline characteristics and biomarkers between the cases and control group [Median (Q1-Q3)] n=124.

Characteristics	Case (n=62)	Control (n=62)	p-value

	Median (Q1-Q3)	Median (Q1-Q3)	
Age (years)	26.5 (24.7-30.0)	28.0 (25.0-32.0)	0.186
BMI (kg/m^2^)	28.0 (21.8-30.0)	27.0 (21.7-29.0)	0.847
Vitamin-B12 (pg/ml)	288.5 (216.0-443.0)	312.0 (243.7-382.7)	0.295
Folic acid (ng/ml)	6.5 (3.5-9.4)	8.1 (6.3-9.3)	0.022*

* p-value calculated by using Mann-Whitney U test BMI: Body mass index.

No significant correlation was observed between homocysteine with Vitamin-B12 (r=0.124, p=0.338) and folic acid (r=0.067, p=0.606) in the RPL group Fig.1A and 1B. The mean value of homocysteine was slightly higher in RPL patients in comparison with that of the control group Fig.2.

**Fig.1A:**
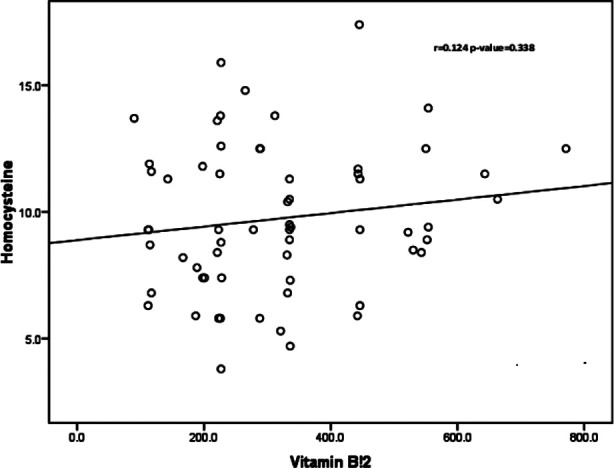
Spearman’s correlation between serum homocysteine and Vitamin-B12 in RPL group.

**Fig.1B:**
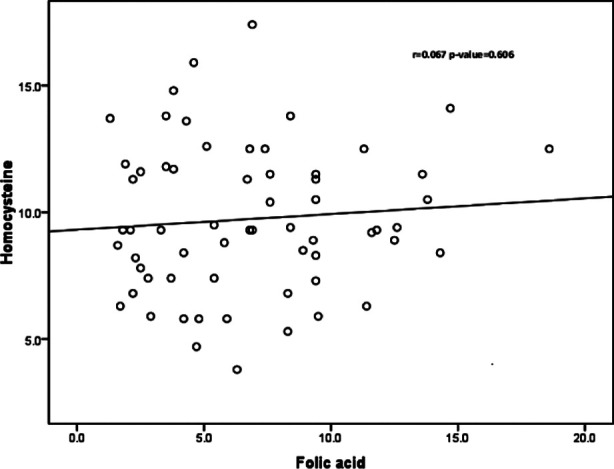
Spearman’s correlation between serum homocysteine and folic acid in RPL group.

**Fig.2:**
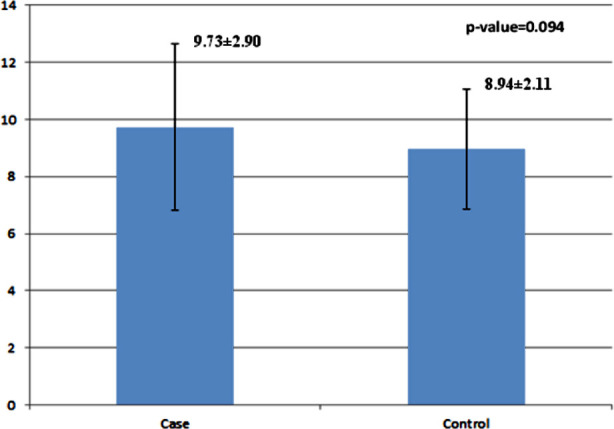
Mean ± SD of homocysteine in studied groups.

Regarding homocysteine, 4 (6.5%) cases were raised the cut off level (14 umol/L), as compared to 1 (1.6%) control. Concerning vitamin-B12, 15 (24.2%) cases and 2 (3.2%) controls were plasma levels below the cutoff value (174 pg/ml). With regards to folic acid, 11 (17.7%) cases and 1 (1.6%) control were below the cutoff level (2.6 ng/ml). There was significant association of vitamin-B12 and serum folate levels with RPL (p<0.05) while age group, BMI group and homocysteine level were not significantly associated with RPL (p>0.05) (Table-II).

**Table-II:** Association of factors with RPL (n=62 each, %).

	n=124	Case n(%)	Control n(%)	χ^2^/p
***Age groups***				
	≤ 28	37 (59.7)	33 (53.2)	0.469
	>28	25 (40.3)	29 (46.8)	
***BMI (kg/m2)***				
	Non-Obese	26 (41.9)	23 (37.1)	0.714
	Obese	36 (58.1)	39 (62.9)	
***Homocysteine (μmol/L)***				
	Normal (<14)	58 (93.5)	61 (98.4)	0.365
	Raised (≥14)	4 (6.5)	1 (1.6)	
***Vitamin-B12 (pg/ml)***				
	Low (<174)	15 (24.2)	2 (3.2)	0.001*
	Normal (174-878)	47 (75.8)	60 (96.8)	
***Folic acid (ng/ml)***				
	Low (<2.6)	11 (17.7)	1 (1.6)	0.004*
	Normal (2.6-12.7)	51 (82.3)	61 (98.4)	

* p-value calculated by using Pearson’s chi-square test and Fisher’s exact test.

## DISCUSSION

The development of RPL is influenced by various contributing factors. However, more than half of RPL cases have unknown etiology which is a source of frustration for both patients and clinicians. Hyperhomocystinemia is one of the risk factors for thrombosis. Several studies have been conducted to find a potential correlation between hyperhomocysteinemia and RPL, but studies have reported varying results in different population groupings.^10^ Unfortunately, no such study has been conducted in Pakistan, a part of world with different racial and ethnic backgrounds. Our study was initiated with the assumption that there is a relationship between serum homocysteine, folic acid and vitamin B12 levels and RPL.

In our research, we observed elevated level of homocysteine in cases as compared to controls, however the difference did not reach the significance level. Creus et al. also reported insignificant relationship of homocysteine with RPL.^11^ However, this finding is in contrast to a study conducted in South East Asia, which proposed that hyperhomocysteinemia serves as an independent risk factor for RPL.^12^ Nutritional insufficiencies of vitamin-B12 or folate, or impaired function of intermediates in the homocysteine metabolic pathway, may lead to elevation in plasma homocysteine levels.^13^ Malnutrition or malabsorption of vitamin-B12 or folate are also important factors that can result in increased concentration of homocysteine in the blood.^14^ Moreover, mutation in the gene encoding methylene tetrahydrofolatereductase (MTHFR) enzyme, decreases enzyme activity resulting in hyperhomocysteinemia.^8^

People with mild impairments in folate metabolism rarely express any complaints concerning their own health; however, they may present with complications in pregnancy since folate metabolism plays a fundamental role for the maintenance of healthy pregnancy.^14^ Folate and vitamin-B12 are both equally important in this process. In the cytosol, conversion of 5-methyl tetrahydrofolate (5-MTHF) to tetrahydrofolate (THF) occurs during the re-methylation of homocysteine to methionine, which is assisted by Vitamin-B12. THF is essential for de-novo biosynthesis of deoxy thymidylate monophosphate (dTMP) in the nucleus,^15^ the latter being the only source of thymidine for DNA synthesis and repair.

Our study found significant difference in folic acid levels between both the study groups: cases having a lower level of folate as compared to the control group. This is similar to the findings reported by a study conducted in Italy 16 and a meta-analysis; 7 however, the clinical study conducted in this meta-analysis has conflicting results due to the fact that all the study participants were taking folic acid supplements.^7^,^16^ Low plasma folate levels are linked to miscarriages, as folic acid may have an important role in quality of oocyte and developing embryo.^17^

Folic acid and vitamin-B12 both are essential in homocysteine metabolism: the former is the substrate, and the latter is the co-factor for MTHFR. In our study, vitamin-B12 levels were decreased in cases as compared to controls, but the difference was insignificant. Our finding is in line with a previously conducted study,^11^ however, another study conducted in Egypt reported a significant difference in vitamin-B12 levels between RPL cases and controls.^18^ It has been suggested previously that vitamin-B12 deficiency can be more accurately diagnosed with serum measurements of the bioactive vitamin-B12 fraction methylmalonic acid (MMA) and holotranscobalamin (holoTC), but they are not clinically validated.^19^,^20^

By applying the normal cutoff values mentioned in Table-II, our results showed a significant association of serum vitamin-B12 and folic acid with RPL. Our findings are consistent with other studies reported association of RPL with low level of serum vitamin B12 and folic acid,^21^ while observations of El-Kadi et al and others concluded that high homocysteine, low vitamin-B12 and low folic acid levels are associated with RPL.^22^, ^23^ Hyperhomocysteinemia is an indicator of oxidative stress and balanced homocysteine level is crucial for both maternal and fetal health. Low level of folic acid and vitamin B12 may contribute for the etiology of first trimester unexplained RPL. It may be essential to determine serum level of these components in women of RPL. Folic acid and vitamin B12 support may be beneficial in the RPL cases.

### Limitations

It includes small sample size and participants mostly belonging to low socioeconomic group, the parameters assessed in our research will be beneficial for patients with RPL. More studies should be conducted on a larger scale, including a larger number of RPL patients from diverse socioeconomic background, to substantiate our findings. It is also important to document nutritional intake and genetic polymorphism studies, which will further help to elaborate one of the risk factors of RPL.

## CONCLUSION

Our study shows that reduced level of folic acid and vitamin-B12 is significantly associated with recurrent pregnancy losses. Homocysteine was marginally raised in our set of RPL patients; however, the difference was not significant. It is recommended that folic acid and vitamin-B12 supplementation is likely to be beneficial in improving pregnancy outcomes in recurrent pregnancy loss patients. Moreover, early follow-up and treatment may lessen the disease burden.

### Authors Contribution:

**ER:** Conceived, designed, did data collection and manuscript writing & editing of manuscript, is responsible for integrity of research.

**AA:** Did review and final approval of manuscript

**RA:** Did data collection.

**HFW:** Did review statistical analysis.
